# Supplementary material for: Validation of the Keen Eye computer-based method for diagnosing visual neglect using a dual-task paradigm
Source: PLoS One. 2025 Oct 27;20(10):e0323832. doi: 10.1371/journal.pone.0323832 (PMC12558459; doi:10.1371/journal.pone.0323832)

DATE:

NEGLECT SYNDROME DIAGNOSTIC FORM

FULL NAME:

DIAGNOSIS:

DATE OF BIRTH:

EDUCATION:

CURRENT EMPLOYMENT:

HANDEDNESS, FAMILIAL LEFT-HANDEDNESS:

HEALTH COMPLAINTS:

CONTACT ACCESSIBILITY:

INTEREST IN THE STUDY:

CRITICISM [1]:

DYNAMIC CHARACTERISTICS:

ADDITIONAL INFORMATION:

---

[1] Cognitive, emotional criticism of oneself, of mistakes made, of the examination situation.

CBS - Catherine Bergego Scale

№	Question	0	1	2	3
**1**	Forgets to groom or shave the left part of his/her face				
**2**	Experiences difficulty in adjusting his/her left sleeve or slipper				
**3**	Forgets to eat food on the left side of his/her plate				
**4**	Forgets to clean the left side of his/her mouth after eating				
**5**	Experiences difficulty in looking towards the left				
**6**	Forgets about a left part of his/her body (e.g. forgets to put his/her upper limb on the armrest, or his/her left foot on the wheelchair rest, or forgets to use his/her left harm when he/she needs to)				
**7**	Has difficulty in paying attention to noise or people addressing him/her from the left				
**8**	Collides with people or objects on the left side, such as doors or furniture (either while walking or driving a wheelchair)				
**9**	Experiences difficulty in finding his/her way towards the left when traveling in familiar places or in the rehabilitation unit				
**10**	Experiences difficulty finding his/her personal belongings in the room or bathroom when they are on the left side				

Total score: __/30
0 – no neglect, 1 – mild neglect, 2 – moderate neglect, 3 – severe neglect

Bells Test-Demonstration

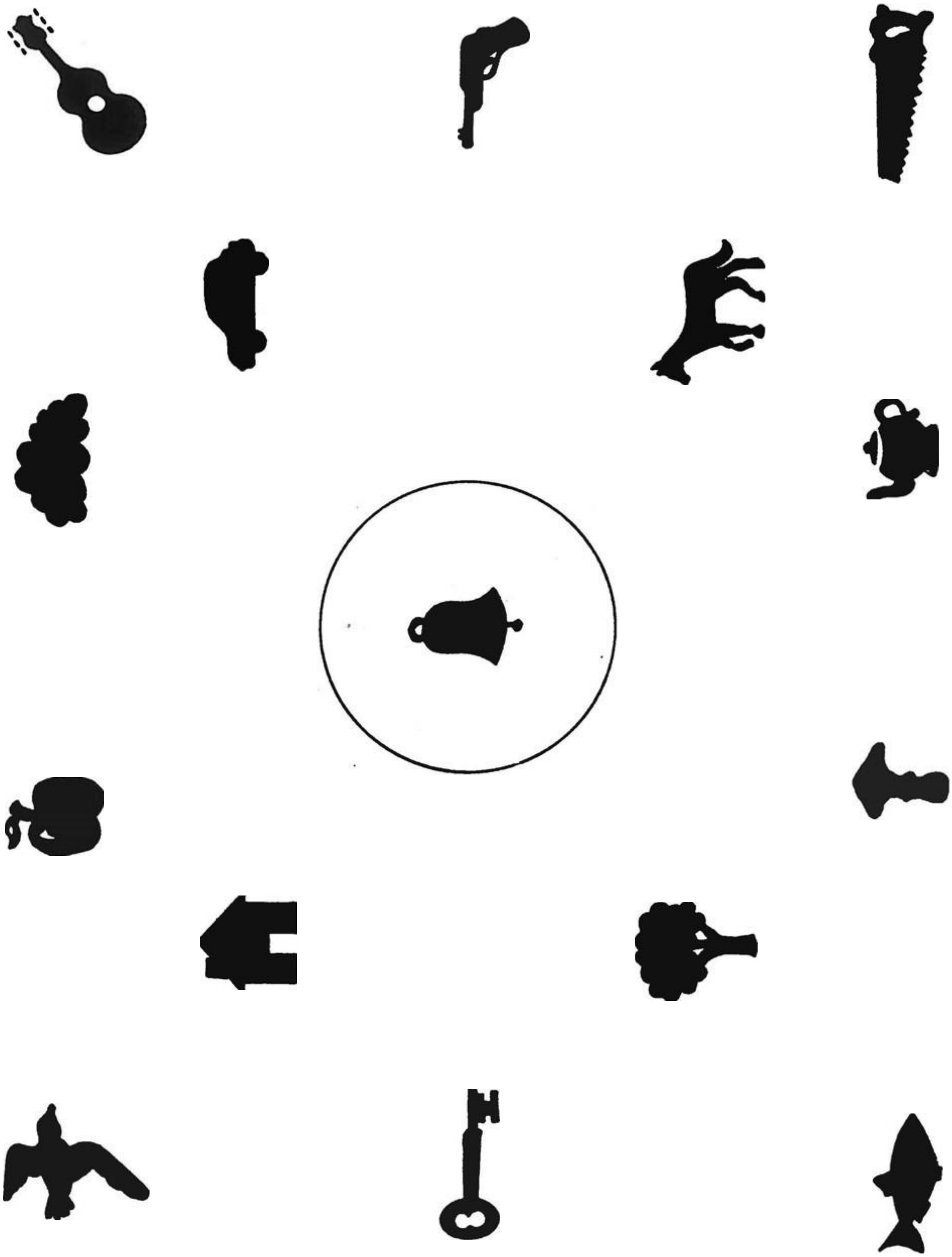

Bells Test-Client

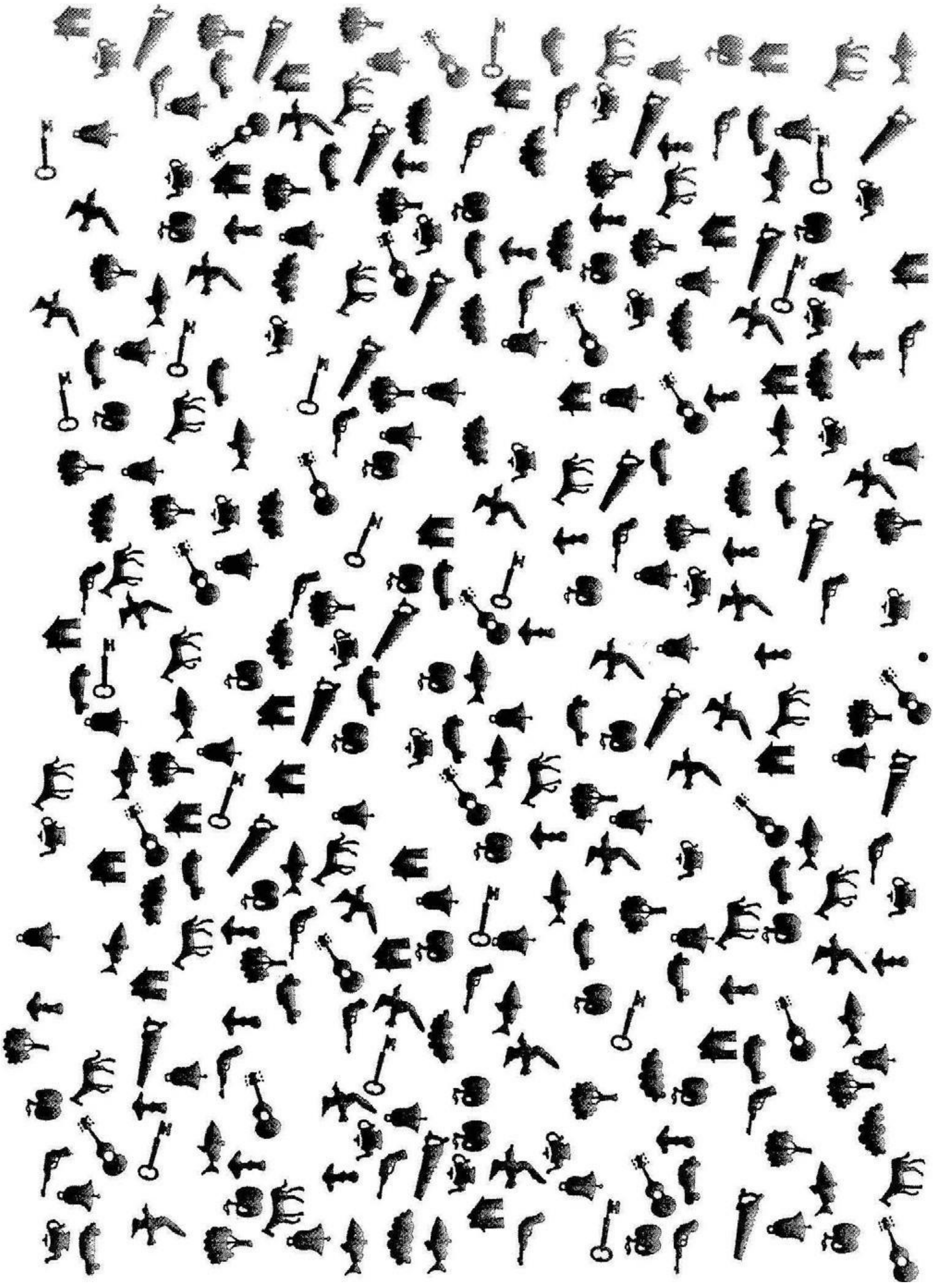

Bells Test - Scoring sheet

Total number of bells circled:	/ 35	Number of Left omissions:	
Realisation time (minutes):		Number of Right omissions:	
Time taken to complete test (minutes):			

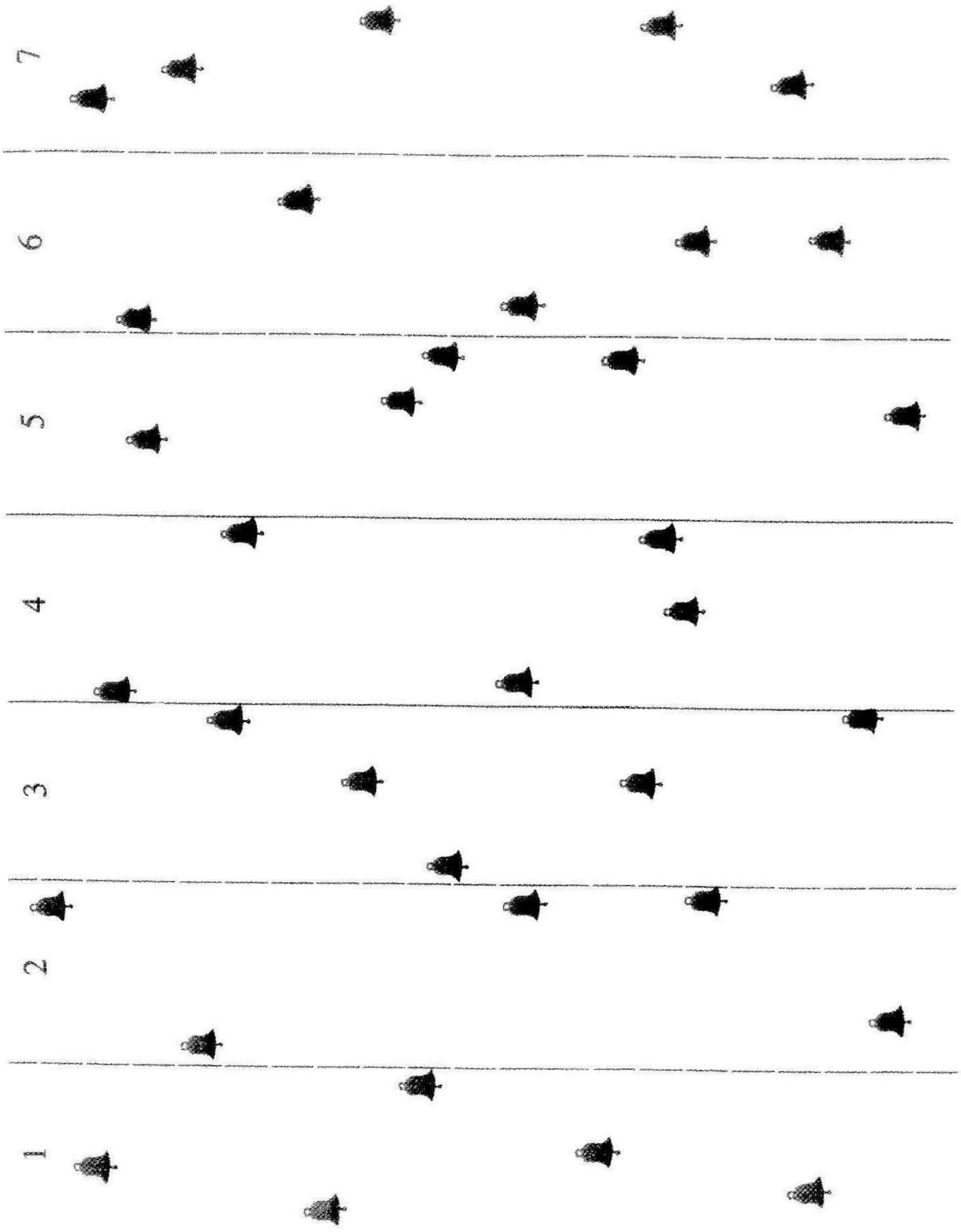

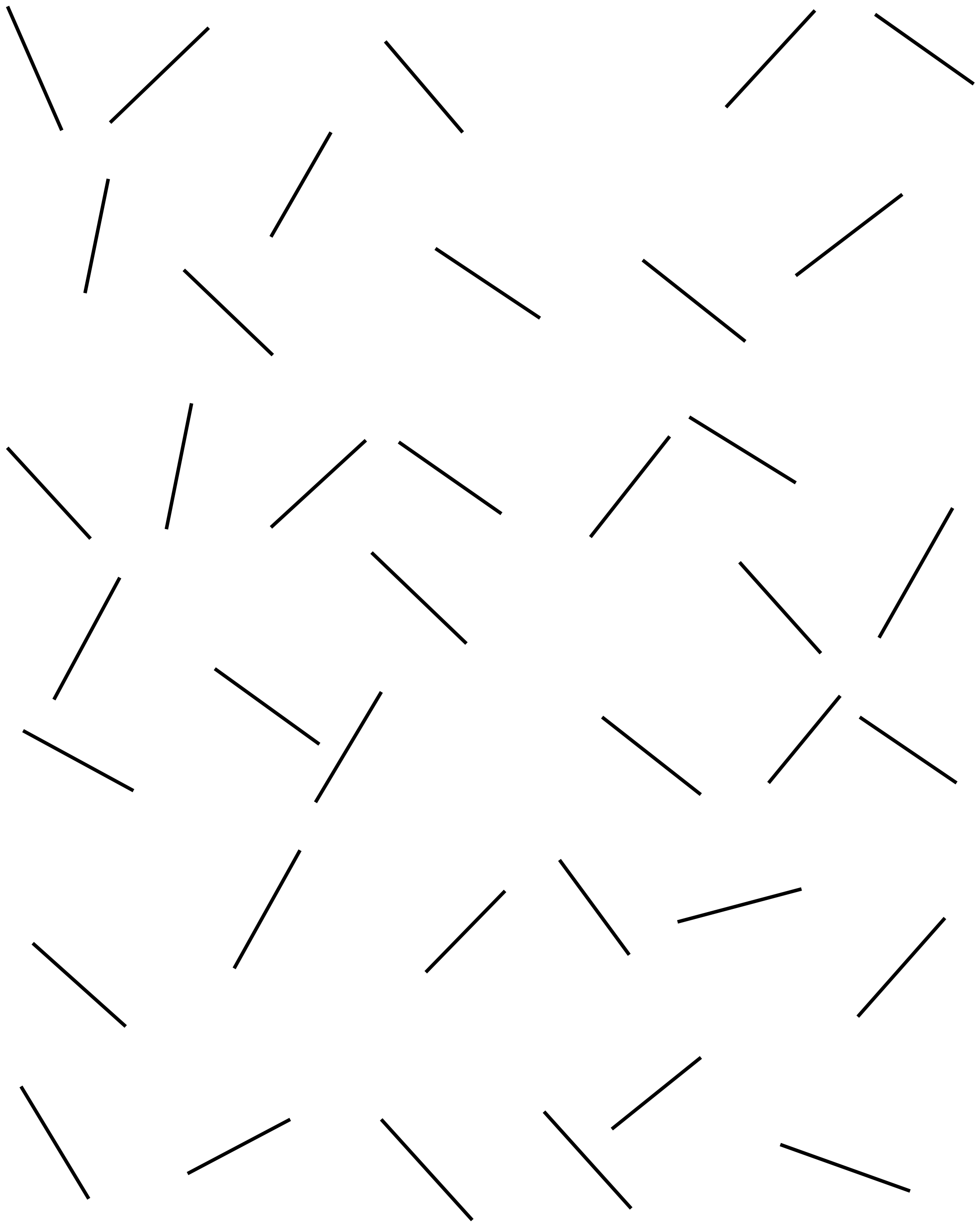

Frontal Assessment Battery (FAB)

Components	Scoring	Commentary	Score
1. Conceptualization)	0-3	An answer that contains a categorical generalisation (‘This is fruit’) is considered correct. If the patient has difficulty or gives a different answer, they are told the correct answer. Each categorical generalisation is scored 1 point.<br>1) What do an apple and a pear have in common?<br>2) What do a coat and a jacket have in common?<br>3) What do a table and a chair have in common?	
2. Lexical Fluency	0-3	“Say as many words as you can beginning with the letter 'C,' (S) any words except surnames or proper nouns.” The time allowed is 60 seconds. **Result:** more than 9 words - 3 points, 7-9 - 2 points,<br>4-6 - 1 point, less than 4 - 0 points.	
3. Motor Series	0-3	A series of three movements:<br>• Fist (placed horizontally, parallel to the table surface)<br>• Edge (hand placed vertically on the medial edge)<br>• Palm (the hand is placed horizontally, palm downwards).<br>1) The patient does it themselves. Hints are not allowed.<br>2) The patient repeats after the examiner<br>3) The patient does with repetition and narration<br>**Result:** correct performance of the first series - 3 points, the second series - 2 points, the third series - 1 point, failure to perform - 0 points.	
4. Conflicting Instructions	0-3	**Instruction:** "Now I'm going to test your attention. We are going to tap out a rhythm. If I tap once, you tap twice. If I tap twice, you tap only once."<br>Rhythm: 1-1-2-1-2-2-2-1-1-2.<br>**Score:** correct - 3 points, no more than 2 mistakes - 2 points, more than 2 mistakes - 1 point, complete copying of the examiner's rhythm - 0 points.	
5. Go-No Go	0-3	**Instruction:** "Now if I tap once, you don't have to do anything. If I tap twice, you tap only once.<br>Rhythm: 1-1-2-1-2-2-2-1-1-2.<br>**Score:** correct - 3 points, no more than 2 mistakes - 2 points, more than 2 mistakes - 1 point, complete copying of the examiner's rhythm - 0 points.	
6. Prehension Behaviour (Grasp Reflex)	0-3	1. The examiner is seated in front of the patient.<br>2. Place the patient’s hands palm up on their knees.<br>3. Without saying anything or looking at the patient, the examiner brings their hands close to the patient’s hands and touches the palms of both the patient’s hands, to see if he/she will spontaneously take	

		them.<br><br>4. If the patient takes the hands, the examiner will try again after asking them: “Now, do not take my hands.”	

Total score (max=18): ___

6 words

№	Sea [More]	Hay [Seno]	Pipe [Truba]	Lamp [Lampa]	Shadow [Ten’]	Wolf [Volk]	Total number	Additions
1								
2								
3								
4								
5								
6*								

Table Drawing Test

Heterogeneous interference (100 – 7)

Difference:	***93***	***86***	***79***	***72***	***65***	***58***	***51***	***44***
Answers:								

Test of Non-Verbalizable Figures (Memorising)

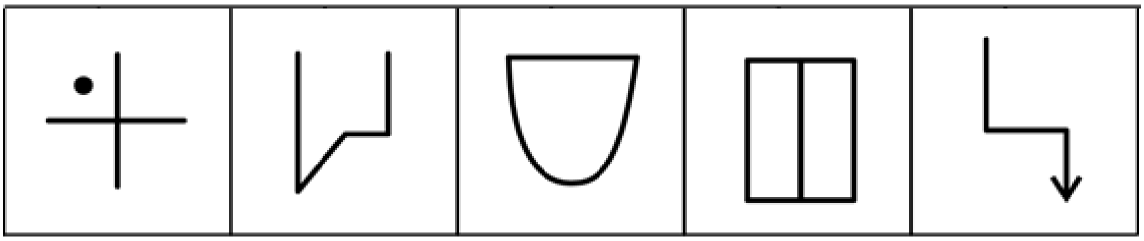

2 sentences

There were apple trees growing in the orchard behind the tall fence. [V sadu za vysokim zaborom rosli yabloni.]
At the edge of the forest, the hunter killed a wolf. [Na opushke lesa okhotnik ubil volka.]

Clock Test, Time: *1) ten to two; 2) 25 minutes past seven; 3) fifteen to three; 4) ten past four; 5) ten to seven*

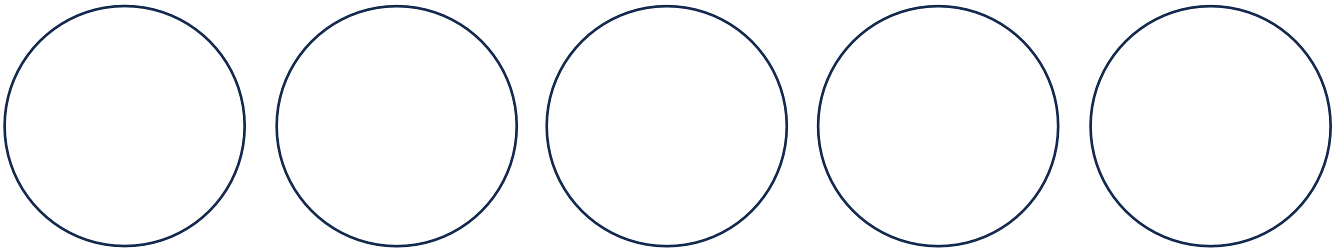

Supplement: S2 File — This supplementary file presents the standardized neuropsychological assessment protocol administered to all participants in the study. The comprehensive test battery was designed to evaluate both neglect-specific deficits and general cognitive functions. For neglect assessment, the protocol included qualitative tests (copying of 5 geometric figures, table drawing, and clock drawing test) alongside quantitative measures (Albert’s Test for visual neglect, Bell’s Test for spatial attention, and Catherine Bergego Scale (CBS) for functional neglect evaluation). Additionally, the protocol incorporated broader cognitive assessments: the Frontal Assessment Battery (FAB) for executive functions, verbal memory tests (memorization of 6 words and 2 sentences), and a serial subtraction task (100−7) for attention and working memory. This multi-domain approach allowed for thorough characterization of neglect patterns while controlling for potential confounding cognitive deficits. The standardized administration ensured consistency across all participants, facilitating reliable comparison of results. (PDF) [file pone.0323832.s002.pdf]
