# Supplementary material for: Validation of the Keen Eye computer-based method for diagnosing visual neglect using a dual-task paradigm
Source: PLoS One. 2025 Oct 27;20(10):e0323832. doi: 10.1371/journal.pone.0323832 (PMC12558459; doi:10.1371/journal.pone.0323832)
Supplement: Attachment — Submitted filename: ReviewerComments.docx [file pone.0323832.s003.docx]

The manuscript presents a relevant contribution to the assessment of visual neglect using a computer-based dual-task paradigm. The rationale is well grounded, and the proposed method has the potential to improve diagnostic sensitivity in both clinical and subclinical populations.

However, there are some theoretical and methodological aspects that would benefit from clarification or further elaboration. Below are specific comments and suggestions aimed at improving the clarity, rigor, and interpretability of the study.

Lines 128-129: Consider clarifying that Lavie’s Load Theory distinguishes between *perceptual load* and *cognitive load* in attentional processing. Specifically, under high *perceptual load*, attentional resources are fully occupied by task-relevant stimuli, thereby reducing the processing of irrelevant distractors. In contrast, under high *cognitive load*, top-down control may be weakened, potentially leading to increased distractor interference.

Lines 163-165: Consider adding that computer-based tasks represent a more promising method for quantifying patients’ performance for several reasons beyond the nature of the tasks themselves. First, they allow for the brief presentation of stimuli and the recording of response latencies with millisecond precision. This makes it possible to detect not only overt failures in processing contralesional space, but also subtle delays in spatial processing. Second, computerized tests can be tailored to the individual’s performance level using adaptive procedures and adjusting parameters such as stimulus duration, contrast, or spatial location until a performance threshold is reached. This reduces the risk of ceiling effects and learning effects. Furthermore, as the authors note, computerized settings facilitate the creation of tasks that engage different levels of attentional resources, up to full resource deployment under the most demanding conditions. While such attentional load can theoretically be induced even without computers, computerized implementation ensures better control, thereby enhancing diagnostic sensitivity and reproducibility.

Consider adding, at the end of the Introduction, a concise overview of the experimental method along with a clear statement of the main hypotheses. This would enhance the logical flow between the theoretical background and the empirical section of the paper. In particular, it would be helpful to briefly justify the choice of the primary task and explain why that specific secondary task was selected to manipulate attentional load.

Lines 182-189: Please clarify how the diagnosis of visual neglect was established in the VN group. It would be important to specify which specific neuropsychological tests were used. In addition, if available, please indicate how right hemisphere damage was confirmed—was neuroimaging (e.g., MRI, CT scan) performed, and were lesion locations documented or analyzed? Furthermore, consider reporting the etiology of the brain lesions (e.g., ischemic or hemorrhagic stroke, tumor), as this information is essential to fully characterize the sample.

Lines 201-206 and lines 229-230: Consider specifying in detail how many an what types of devices were used in administering the task. It is also important to indicate whether participants were seated at a fixed viewing distance from the screen, and if so, how this distance was controlled (e.g., using a chinrest, visual markers, or standardized instructions). Given that the task involves peripheral stimulus localization, variations in screen characteristics or viewing distance could significantly alter the visual angle and perceived eccentricity of stimuli, thereby impacting the task’s diagnostic validity. Providing this information would improve methodological transparency and support replicability across different research and clinical contexts.

Lines 236-240: It would be helpful to clarify how the number of trials was determined. Was this based on previous literature, pilot testing, or a formal power analysis? Given that the number of trials affects both the sensitivity of the task and the potential for participant fatigue, providing a rationale for this choice would strengthen the methodological rigor of the study.

Lines 207-216: In my view, it is important to note that a single-task condition was not included in the study design. Including such a baseline condition would have been usefull to isolate the specific effect of increased attentional load on spatial processing performance. A direct comparison between single-task and dual-task performance would have provided stronger evidence that the observed deficits emerge specifically under multitasking demands.

Lines 236-240: Currently, bilateral targets are only presented in spatially symmetrical positions. While this ensures experimental control, it may limit the ecological validity and diagnostic sensitivity of the task. Consider discussing the possibility of including asymmetrical bilateral target configurations in future versions of the method. Such arrangements could help assess the influence of spatial competition under less predictable and more naturalistic conditions, potentially revealing more subtle forms of extinction or residual neglect.

Lines 318-320: If the control group consists of the 64 patients who did not exhibit visual neglect, please specify this explicitly in the text.

Lines 479-481: Consider citing this recent study: <https://doi.org/10.1038/s41598-025-95717-0>

It shows that increased attentional load affects visual processing in healthy individuals, but without inducing the kind of spatial asymmetries typically observed in patients with neglect. This supports the idea that the asymmetrical effects under high attentional load are pathological rather than task-induced, and would strengthen the theoretical framework of your discussion.
