# Supplementary material for: Validation of the Keen Eye computer-based method for diagnosing visual neglect using a dual-task paradigm
Source: PLoS One. 2025 Oct 27;20(10):e0323832. doi: 10.1371/journal.pone.0323832 (PMC12558459; doi:10.1371/journal.pone.0323832)
Supplement: Attachment — Submitted filename: Response to Reviewers.docx [file pone.0323832.s005.docx]

**Manuscript PONE-D-25-18591**

**Response to Reviewers**

Dear Dr. Contemori,

Thank you for giving us the opportunity to submit a revised version of our manuscript "Approbation of the Keen Eye Computer-Based Method for Diagnosing Visual Neglect" for publication in PLOS ONE. We sincerely appreciate the time and effort that you and the reviewers dedicated to evaluating our work and providing constructive feedback. We are grateful for the valuable comments, which have helped us improve the clarity, rigor, and impact of our study.

We have carefully addressed all the points raised by the reviewers. Below, we provide a point-by-point response to each comment. Changes in the manuscript are highlighted in the "Revised Manuscript with Track Changes" file. All page and line numbers refer to this version.

# **Editor’s Comments**

1. Several critical issues were identified—particularly with regard to the clarity and completeness of the sample characterization and the methodological description of the task.

**Author Response:** *We have added a detailed description of patient characteristics in the Participants section (Lines 234–248), including information on etiology (ischemic vs. hemorrhagic), time since onset, stage of recovery (subacute or chronic), and neuroimaging confirmation of right-hemisphere lesions. We also clarified the process of clinical diagnosis of visual neglect (Lines 249–278).*

2. Specific concerns were raised regarding the detailed reporting of patient characteristics, the operationalization of diagnostic criteria, and the transparency of the task’s implementation and statistical analyses.”

**Author Response:** W*e addressed this by:*

- *Specifying the exact clinical and neuropsychological criteria used to establish a diagnosis of neglect (Lines 249–278).*
- *Adding detailed descriptions of task design and implementation, including visual angle calculations, stimulus positioning, trial count rationale, and device specifications (Lines 288–362).*
- *Including a subsection on the decision tree modeling approach and cross-validation settings (Lines 484–513), and defining all relevant metrics (gini index, precision, recall, F1-score).*

3. Questions emerged concerning the novelty of the method in relation to previous work, which should be adequately credited, as well as some theoretical and interpretative aspects of the findings.

**Author Response:** *We clarified in the Introduction (Lines 126–134) that our dual-task approach is built on earlier theoretical and experimental traditions. In the Discussion (Lines 643–661), we explicitly differentiate the Keen Eye method from prior studies, particularly those by Bonato et al., and explain how our work extends this foundation in terms of design, clinical applicability, quadrant-level analysis, and integration of clustering-based diagnostics.*

4. We note that your Data Availability Statement is currently as follows: [All relevant data are within the manuscript and its Supporting Information files.]”

**Author Response:** *The Data Availability Statement has been updated in accordance with the requirements. We now confirm that all raw data necessary to replicate the results of the study are included in the manuscript and its Supporting Information files. Specifically:*

- *The complete dataset used for analyses has been provided as Supplementary File S1.*
- *A detailed legend describing all variable names and acronyms is included in Supplementary File S1 (Sheet 2).*
- *All values behind descriptive and inferential statistics, as well as those used to generate graphs and perform clustering, are fully available.*
- *No ethical or legal restrictions apply to data sharing in this study.*

## **Reviewers' Comments to the Authors:**

**Reviewer #1**

The authors describe the validation of a test capable to assist in the diagnosis and rehabilitation of spatial neglect. The test has been administered to a large cohort of right-hemisphere stroke patients. The method leverages on the presentation of lateralized visual stimuli, which are presented at different eccentricities and possibly the lower/upper visual field. Additionally, the paradigm includes a visual dual-task: patients are asked to report a digit presented at fixation, which is known to increase the sensitivity to lateralized biases. The test has been confirmed to be sensitive to attentional biases and capable to highlight altitudinal neglect.

**Author response:** *We sincerely appreciate the reviewer's recognition of our test's clinical importance and its potential to assist in diagnosing spatial neglect.*

1. In my reading of the paper I found the task extremely similar to that described by Bonato et al. (2010, neuropsychologia). So where does the name “keen eye method” comes from? Are the authors building on previous methods called “keen eye” (not referenced) or suggesting their own? Bonato and colleagues are otherwise duly cited, including a recent preprint (now published in Communications Biology) which appears to have inspired many passages in the introduction.

**Author response:** *Thank you for your observation. We fully acknowledge the foundational role of Bonato et al.’s work in shaping the dual-task approach to diagnosing visual neglect. In response, we clarified in lines 220–222 of the Introduction that Keen Eye is our own term for a newly developed clinical diagnostic tool, inspired by—but not identical to—previous experimental designs. In lines 643–661 of the Discussion, we now explicitly outline the key innovations of the Keen Eye method in comparison to Bonato’s paradigm, including our fixed and vertically extended stimulus layout, the use of asymmetry coefficients, clinical focus, and implementation in open-source software.*

2. My main issue with this manuscript lies in the fact that many aspects are not duly reported. In particular, the sample of patients is not described in several core clinical features. Are the patients tested in the acute, subacute, or chronic stage? This matters a lot. It would be also useful to add information about the different etiologies (i.e., the proportion of ischemic vs hemorrhagic strokes).

**Author response:** *We agree this is crucial information. We have now expanded the description of the patient sample in the Participants section (lines 238–248), clearly indicating the lesion laterality, etiology (ischemic vs hemorrhagic), and stage of recovery (subacute or chronic). In addition, individual-level details for each patient are now included in Supplementary File S1.*

3. Importantly, the behavioral assessment leading to the diagnosis is not properly detailed as well. Later in the text it seems that both the Albert and Bells test were administered, though it remains unclear which one served the reach the diagnosis. Luria’s seminal work is referenced when mentioning that “a preliminary comprehensive neuropsychological examination to assess higher mental functions [72], as well as standardized quantitative assessments of visual neglect”. These standardized tests are what is used to assess convergence and criterion validity, which is why it is very important that all these tests are duly referenced and described early in the text for the benefit of the readers.

**Author response:** *Thank you for pointing this out. We revised the Participants section to clearly state how the diagnosis of visual neglect was made (lines 249–278), based on a comprehensive neuropsychological assessment. We have also added a detailed description of the full neuropsychological battery administered to all participants, now available in Supplementary File S2. This battery included both quantitative and qualitative measures and informed the reference diagnosis used for validating the Keen Eye method.*

Other important points, in no particular order.

4. “Approbation” does not strike me to be a standard term. Consider revising the title and abstract to reflect the content of the paper.

**Author response:** *We appreciate this helpful suggestion. In our native language, the term “approbation” is commonly used to refer to pilot validation of a method prior to formal standardization. However, we recognize that in English-language academic publishing, “validation” is the more appropriate and widely accepted term. Therefore, we have revised the manuscript title and abstract accordingly.*

5. Task parameters are given in terms of centimeters from the screen. Since it appears that there may be variability in the equipment used (different screen sizes and OS are mentioned), and the distance of the patients from the screen is not reported, I would advise to use degrees of visual angle as well.

**Author response:** *Thank you for this important recommendation. We have clarified the technical setup in the Materials section, including screen size, laptop model, and operating system (lines 288–294). We now report stimulus eccentricity both in centimeters and in degrees of visual angle, assuming a typical viewing distance of 60 cm (lines 343–346, 361–362). We also specify how patient distance to the screen was monitored (line 359-360) and acknowledge that formal distance fixation (e.g., chinrest) was not used due to clinical feasibility constraints.*

6. Several measures in the statistical analyses are not defined or lack details: gini index, precision, recall, F1 score, all should be briefly defined for the readers. The crossvalidation setup is not explicit: how many folds were used? Which measure was used to evaluate cv performance?

**Author response:** *Thank you for this important remark. A clear understanding of these metrics is essential for interpreting model performance. We have added concise definitions for the Gini index, precision, recall, and F1 score in lines 484–496 to help readers unfamiliar with these terms.*

*We also clarified the cross-validation setup: it was implemented using the rpart.control function with the xval parameter set to 10, thereby creating 10 equal-sized folds (see lines 499–513). To ensure model stability and interpretability, we specified several additional tree-growing parameters:*

- *minsplit = 20: minimum number of observations required for a split;*
- *minbucket = 7: minimum observations in terminal nodes;*
- *cp = 0.01: complexity parameter for pruning.*

*These parameters were chosen to balance model generalizability and overfitting risk, and the complexity parameter (cp) was used as the main criterion for evaluating CV performance.*

7. Figure captions could be more descriptive. For example, figures 4,5,6 report the decision tree from three different models, but it is not spelled out the specificity of each. It would be useful to have reported the starting variables and the models’ goals (e.g., to maximize specificity) in the captions as well.

**Author response:** *We fully agree that enhanced figure captions would improve clarity. Therefore, we have revised the captions for Figures 7, 8, and 9 (corresponding to Figures 4, 5, and 6 in the previous version of the manuscript) to include the model objective, list of input variables, and performance focus (e.g., balanced specificity or sensitivity or maximized sensitivity). These changes make it easier for readers to understand the logic and goals of each classification tree.*

8. The authors write “The Keen Eye method identified LSB ≥ 12 as the threshold for diagnosing neglect, as determined by the classification tree method.” However, Figure 5 reports LSB <14. Please clarify.

**Author response:** *Thank you for pointing this out. You are absolutely correct—this was a typographical error. The correct threshold determined by the classification tree is LSB <14, not ≥12. We have corrected this in the revised manuscript, specifically in line 585.*

9. As the authors acknowledge, one limitation is that the test can be long and tiring. Can the authors identify a subset of the original positions on screen such that enough sensitivity for the diagnosis is given, but with fewer trials? For example, would it be enough to administer the most eccentric positions in both the lower and upper visual fields?

**Author response:** *Thank you for raising this practical and clinically important question. We fully agree that test duration impacts patient performance and usability in real-world settings. In response, we have added a subsection titled "Diagnostic efficiency of individual target positions" to the Results section (lines 559–581), in which we evaluated the sensitivity and specificity of each stimulus location. While we are not yet ready to formally exclude any positions from the task, we discuss in this section several target locations that may be considered for future task reduction based on their diagnostic utility. These findings lay the groundwork for a more concise version of the test.*

10. Only dual-task conditions were administered. Do the authors consider useful to administer conditions without additional demands, that is a purely spatial task? For example, the authors suggest that the paradigm could be more sensitive, and identify as neglect more patients than the conventional testing. Would these patients show difficulties without multitasking? Would the definition of the clusters change when the original variables also include single task conditions?

**Author response:** *This is an excellent methodological point, and we appreciate the reviewer’s attention to the paradigm structure. We intentionally did not include a single-task condition because the primary aim of this project was to design a clinically usable diagnostic tool, prioritizing brevity, clarity, and standardization (see lines 308–316). We acknowledge, however, that the absence of a baseline single task condition limits interpretability regarding the specific effect of attentional load. We have now explicitly stated this in the Limitations section (lines 831–836). Regarding the potential impact on cluster composition: we hypothesize that including single-task performance may reduce the distinction between clusters 2 and 3 (both representing neglect), as these groups already do not differ significantly on general executive functioning (as assessed by the FAB). However, this remains speculative and would need to be empirically tested in future work.*

**Reviewer #2**

The manuscript presents a relevant contribution to the assessment of visual neglect using a computer-based dual-task paradigm. The rationale is well grounded, and the proposed method has the potential to improve diagnostic sensitivity in both clinical and subclinical populations.

However, there are some theoretical and methodological aspects that would benefit from clarification or further elaboration. Below are specific comments and suggestions aimed at improving the clarity, rigor, and interpretability of the study.

**Author response:** We thank the reviewer for this thoughtful and constructive feedback. We greatly appreciate the recognition of our work’s potential clinical relevance and have addressed all points in detail below.

1. Lines 128-129: Consider clarifying that Lavie’s Load Theory distinguishes between perceptual load and cognitive load in attentional processing. Specifically, under high perceptual load, attentional resources are fully occupied by task-relevant stimuli, thereby reducing the processing of irrelevant distractors. In contrast, under high cognitive load, top-down control may be weakened, potentially leading to increased distractor interference.

**Author Response:** *Thank you for the suggestion. We have revised the relevant section to clarify that Lavie’s Load Theory distinguishes between perceptual and cognitive load and that these two types of load exert opposite effects on distractor processing. The clarification has been added to the Introduction, lines 148–158.*

2. Lines 163-165: Consider adding that computer-based tasks represent a more promising method for quantifying patients’ performance for several reasons beyond the nature of the tasks themselves. First, they allow for the brief presentation of stimuli and the recording of response latencies with millisecond precision. This makes it possible to detect not only overt failures in processing contralesional space, but also subtle delays in spatial processing. Second, computerized tests can be tailored to the individual’s performance level using adaptive procedures and adjusting parameters such as stimulus duration, contrast, or spatial location until a performance threshold is reached. This reduces the risk of ceiling effects and learning effects. Furthermore, as the authors note, computerized settings facilitate the creation of tasks that engage different levels of attentional resources, up to full resource deployment under the most demanding conditions. While such attentional load can theoretically be induced even without computers, computerized implementation ensures better control, thereby enhancing diagnostic sensitivity and reproducibility.

**Author Response:** *Thank you for this valuable comment. We fully agree and have incorporated this elaboration into lines 103–116 of the Introduction. We emphasize the advantages of computer-based testing, including brief and precise stimulus presentation, individual task adaptability, avoidance of ceiling effects, and fine control over attentional load.*

3. Consider adding, at the end of the Introduction, a concise overview of the experimental method along with a clear statement of the main hypotheses. This would enhance the logical flow between the theoretical background and the empirical section of the paper. In particular, it would be helpful to briefly justify the choice of the primary task and explain why that specific secondary task was selected to manipulate attentional load.

**Author Response:** *We appreciate this suggestion. We have revised the subsection Dual Task Paradigm in Introduction to include a clearer overview of the experimental method and key theoretical foundations. Additionally, we added a set of explicit hypotheses and justification for the experimental design in lines 220–230 of the Introduction.*

4. Lines 182-189: Please clarify how the diagnosis of visual neglect was established in the VN group. It would be important to specify which specific neuropsychological tests were used. In addition, if available, please indicate how right hemisphere damage was confirmed—was neuroimaging (e.g., MRI, CT scan) performed, and were lesion locations documented or analyzed? Furthermore, consider reporting the etiology of the brain lesions (e.g., ischemic or hemorrhagic stroke, tumor), as this information is essential to fully characterize the sample.

**Author Response:** *Thank you for highlighting this important point. We have clarified the diagnostic procedure and criteria for assigning patients to the VN group in the Participants section, lines 249–278. Furthermore, we included a description of the neuropsychological battery used (now available as Supplementary File S2). Right hemisphere lesions were confirmed based on CT imaging (lines 238–239). Etiological information (ischemic vs. hemorrhagic stroke, TBI), lesion stage (subacute vs. chronic), and lesion localization have been reported in lines 238–248 and also documented in Supplementary File S1.*

5. Lines 201-206 and lines 229-230: Consider specifying in detail how many an what types of devices were used in administering the task. It is also important to indicate whether participants were seated at a fixed viewing distance from the screen, and if so, how this distance was controlled (e.g., using a chinrest, visual markers, or standardized instructions). Given that the task involves peripheral stimulus localization, variations in screen characteristics or viewing distance could significantly alter the visual angle and perceived eccentricity of stimuli, thereby impacting the task’s diagnostic validity. Providing this information would improve methodological transparency and support replicability across different research and clinical contexts.

**Author Response:** *Thank you for this important recommendation. We have clarified the technical setup in the Materials section, including screen size, laptop model, and operating system (lines 288–294). We now report stimulus eccentricities in both centimeters and degrees of visual angle, assuming a standardized viewing distance of 60 cm (lines 343–346, 361–362). The patient’s distance from the screen was monitored through instruction and observation (line 359–360), although we acknowledge that formal fixation tools such as a chinrest were not employed due to practical limitations in the clinical setting.*

6. Lines 236-240: It would be helpful to clarify how the number of trials was determined. Was this based on previous literature, pilot testing, or a formal power analysis? Given that the number of trials affects both the sensitivity of the task and the potential for participant fatigue, providing a rationale for this choice would strengthen the methodological rigor of the study.

**Author Response:** *Thank you for this important point. The number of trials per stimulus position was determined based on methodological principles aiming to maximize measurement reliability while minimizing participant fatigue. Each of the 25 target configurations (including both unilateral and bilateral presentations) was shown three times, ensuring that detection failures reflected consistent spatial biases rather than random attentional lapses. This repetition rate was refined through pilot testing, which confirmed that three repetitions yielded stable omission rates without causing excessive fatigue in clinical participants. In terms of spatial layout, target eccentricities were designed to span a range from foveal to peripheral regions: the closest targets subtended approximately 1.4 degrees of visual angle, and the most eccentric targets extended to 13.2 degrees. This spatial spread allowed us to examine both central and peripheral influences on neglect-related omissions, enhancing both ecological validity and diagnostic sensitivity. These clarifications have been added to lines 337–351.*

7. Lines 207-216: In my view, it is important to note that a single-task condition was not included in the study design. Including such a baseline condition would have been usefull to isolate the specific effect of increased attentional load on spatial processing performance. A direct comparison between single-task and dual-task performance would have provided stronger evidence that the observed deficits emerge specifically under multitasking demands.

**Author Response:** *We fully agree that including a single-task condition would have enabled a more direct examination of how dual-task load affects spatial performance. However, our primary aim was to create a clinically usable diagnostic tool that is brief, standardized, and practical for administration in real-world settings. To avoid overburdening patients and prolonging testing sessions, we deliberately excluded a single-task condition (lines 308–316). We now explicitly acknowledge this decision as a limitation in the Discussion section (lines 831–836) and note its implications for the interpretation of load-specific effects.*

8. Lines 236-240: Currently, bilateral targets are only presented in spatially symmetrical positions. While this ensures experimental control, it may limit the ecological validity and diagnostic sensitivity of the task. Consider discussing the possibility of including asymmetrical bilateral target configurations in future versions of the method. Such arrangements could help assess the influence of spatial competition under less predictable and more naturalistic conditions, potentially revealing more subtle forms of extinction or residual neglect.

**Author Response:** *We thank the reviewer for this insightful suggestion. Asymmetrical bilateral target configurations were indeed considered during the development phase. However, due to the need to limit overall testing time and prevent excessive patient fatigue, we opted to include only symmetrical bilateral presentations in this version of the method. We agree that asymmetrical arrangements could enhance ecological validity and improve the detection of subtle extinction effects by introducing more spatial competition. This limitation and future direction have now been added to the Discussion section (lines 836–840).*

9. Lines 318-320: If the control group consists of the 64 patients who did not exhibit visual neglect, please specify this explicitly in the text.

**Author Response:** *Thank you for pointing this out. We have clarified in line 234 that the control group includes the 64 participants who did not meet the criteria for visual neglect. We also revised the Participants section to clearly define the diagnostic criteria used, reinforcing the distinction between neglect and control groups (lines 249–278).*

10. Lines 479-481: Consider citing this recent study: <https://doi.org/10.1038/s41598-025-95717-0>. It shows that increased attentional load affects visual processing in healthy individuals, but without inducing the kind of spatial asymmetries typically observed in patients with neglect. This supports the idea that the asymmetrical effects under high attentional load are pathological rather than task-induced, and would strengthen the theoretical framework of your discussion.

**Author Response:** *We appreciate this excellent suggestion and have incorporated the citation into the Discussion (lines 205–211) as reference [77]. This study indeed supports the argument that spatial asymmetries under high attentional load are pathological rather than task-induced, and it strengthens the theoretical foundation of our interpretation.*

**Reviewer #3**

In a relatively large group study with right hemisphere damaged neurological patients authors tested a computer-based method for detecting omissions of lateralized targets. Overall the method seems to have successfully detected contralesional omissions in the group of neglect patients. My main criticisms are related to the very close resemblance with previous studies and to the ambiguity in diagnostic criteria, in the analysis and in the purpose of the study.

**Author Response:** *We appreciate the reviewer’s careful reading and constructive comments. Below we respond to each point in detail.*

.

1. The logic behind the method as well as its implementation are very close if not almost identical to the methods that have been developed by the research group in Padova for the past 15 years (starting from Bonato et al., Neuropsychologia 2010 and Neuropsychology 2012). The resemblance is striking with respect to all aspects of the study as it includes the presence of a visual dual-task with a central digit to be reported along with lateralized targets (ipsi, contra or bilateral) whose detection accuracy is the dependent variable. The similarity extends to the clustering logic and to the interpretation in terms of compensatory strategies. While authors fully acknowledge previous studies the fact that their approach is virtually a replication of these previous studies should in my view be made explicit in the background section as well as for all the relevant sections with high resemblance (methods, analysis, discussion). Although to a lesser extent, also the idea of providing a name for the testing somehow clashes with the idea that the same method has been described in about ten previous papers and seems to result in indirect shadowing of previous studies. The aspects of novelty seem to be related to the spatial uncertainty and to the possibility to test together vertical and horizontal but paradoxically these new features are not tested explicitly nor emphasized much.

**Author response:** *We fully acknowledge that our method builds upon the dual-task paradigm originally developed by Bonato and colleagues, whose contributions to the field are substantial. We have also added a clarification that the dual-task approach is rooted in a broader tradition of earlier attentional research paradigms, including classic studies on resource allocation and multitasking. This addition can be found in the revised manuscript (lines 126–134), where we acknowledge the historical foundations of the dual-task method and position our work as an extension of this established framework. As suggested, we have made this connection more explicit in the Introduction section (lines 220–227), where we now state that the Keen Eye method extends this foundational approach. However, we also respectfully emphasize that our method introduces important innovations beyond prior work. These include:*

- *A fixed, balanced spatial configuration covering both horizontal and vertical axes, enabling quadrant-level analysis (including detection of altitudinal biases);*
- *A diagnostic, rather than purely experimental, aim with standardized administration, thresholds, and interpretation;*
- *Novel asymmetry coefficients designed for dimensional assessment of neglect severity;*
- *Open-source implementation in PsychoPy, facilitating clinical adoption and reproducibility;*
- *Inclusion of features like pausing, resuming, and adapting for patient fatigue in clinical environments.*

*These points are now explicitly described in the Discussion (lines 643–661) and supported by examples throughout the revised manuscript. We also clarified that the name “Keen Eye” reflects a new clinical tool rather than an existing paradigm, and we have revised the phrasing to avoid any unintended overshadowing of prior studies.*

2. As for the availability of the data, many crucial aspects are missing including the etiology of the lesions (vascular only?) time from stroke and, most important, a clear operationalized set of criteria for neglect diagnosis. There is reference to two paper and pencil tests but these data are not reported nor it is clear whether all patients underwent testing with these two tests (only). More specifically, was the diagnosis for left neglect, for right neglect or for altitudinal neglect ?

**Author response:** *Thank you for highlighting this important issue. We have thoroughly revised the Participants section to provide a comprehensive description of the sample. Specifically, we now specify lesion etiology (ischemic vs. hemorrhagic), verified by CT imaging (lines 238–248), we report the time since onset, noting that all participants were in the subacute or chronic phase (no acute cases), we added a table and provided supplementary data (File S1) with detailed clinical information. We clearly describe how the diagnosis of left-sided visual neglect was established based on a combination of clinical neuropsychological assessment, including both the Albert and Bells tests, as well as a comprehensive neurological examination (lines 249–278). To further enhance transparency, we added a separate supplementary file (S2) describing the complete neuropsychological battery administered to all participants.*

3. As for the digital task, there is no indication about whether a response to the central digit was performed. If yes the handling of wrong responses should be described. If no, the ratio should be made explicit.

**Author response:** *Thank you for raising this important point. We have now clarified this in the Methods section (lines 385–394). Participants were instructed to report the digit presented at central fixation during each trial. Incorrect responses were recorded, including both misidentification and omissions (e.g., saying “nothing” or “something flashed, but I don’t know what”). These were aggregated into three variables—NIR (errors during right-target trials), NIL (errors during left-target trials), and NIB (errors during bilateral-target trials). However, we did not further analyze these variables in the current manuscript, as the central digit task served primarily to impose attentional load rather than to diagnose neglect. This choice was made to minimize overfitting and Type I errors due to the large number of derived metrics. This limitation is now acknowledged in the Limitations section (lines 841–847), and we note that future studies may explore these variables more thoroughly.*

4. The ultimate aim of the study seems to be missing. The text refers multiple times to “high diagnostic accuracy” but this concept does not seem to have been fully operationalized. I think it would be clearer to explicitly diagnostic criteria for the different approaches (clinical vs computer based). Moreover, the task does not include a single task condition and therefore does not allow quantifying the effect of multitasking: From this point of view the statement about the presence of general impairments seems a bit far-fetched.

**Author response:** *We appreciate this opportunity to clarify. The diagnostic aim of our study was to develop a sensitive, quantitative, and clinically usable method for identifying visuospatial neglect. Clinical diagnosis was established through standard neuropsychological assessment (detailed in lines 249–278). The Keen Eye task's diagnostic thresholds were derived using classification tree models, as described in the Results section. The most reliable predictors were LSB ≥ 14 (Model 2) and KA_all ≥ 0.17 (Model 3), as described in lines 514–529. Both models showed similar accuracy, but KA_all is independent of the number of target presentations, enhancing robustness. We intentionally excluded a single-task condition to reduce patient burden and task duration. This decision is now clearly justified in the Methods (lines 308–316) and its implications are discussed as a limitation (lines 831–836). While we hypothesize—based on prior literature—that dual-task demands amplify neglect symptoms, we acknowledge that our design cannot isolate these effects directly.*

5. Strictly speaking LSB is an index of extinction not of neglect, and ideally should be corrected with respect to LSU. It is acceptable to keep it the way it is but this limitation should be spelled out.

**Author response:** *We agree and have clarified this point in the Discussion (lines 666–678). LSB (left stimuli skipped in bilateral trials) is more sensitive to extinction than classic neglect, particularly since bilateral trials directly engage competitive attentional mechanisms. Because the total number of trials differs between LSU (21) and LSB (33), we note that direct comparison is limited. We retained LSB as a practical diagnostic marker due to its high sensitivity to subtle deficits, especially in mild or recovered cases. However, we now explicitly discuss the limitation of using LSB in isolation and recommend that future research explore extinction-corrected indices or normalize LSB relative to LSU to disentangle these effects.*

6. Considering the presence of several tests (supplementary materials) I think it would be better to explicity focus on the idea that neglect from many points of view can be conceived within a continuum of omissions.

**Author response:** *Thank you for this valuable suggestion. We agree that spatial neglect should increasingly be conceptualized as a graded phenomenon rather than a binary condition. In the revised manuscript, we highlight this dimensional perspective more explicitly in the Discussion (lines 695–700). However, we also note that for practical clinical use, it is essential to define threshold criteria for identifying patients who may benefit from targeted rehabilitation. Therefore, while our approach acknowledges the spectrum nature of neglect, we also provide binary classification markers derived from decision-tree models (e.g., LSB ≥14 or KA_all ≥0.17) to guide clinical interpretation. Your comment also prompted us to revisit a striking aspect of our data: the elevated number of omissions in far-right positions, not only among neglect patients but also in the control group (patients without neglect). This calls into question the assumption of a simple linear gradient of leftward omissions and supports a more complex, non-linear view of spatial bias. We address this further in response to your next point and in the revised Discussion.*

7. There are several acronyms (about 10). Due to their presence Table 3 is very difficult if not impossible to be understood. The full dataset seems full of relevant information yet the current format of table and the graphs do not seem to allow fully appreciate this info. Finally (Again in Table 3) variables 1 to 14, denoting the different positions, could be made more easy to visualize if ordered left to right.

**Author response:** *We appreciate this feedback and have taken several steps to improve clarity:*

- *In Tables 3 and 5, we reorganized position variables 1–14 in a left-to-right, top-to-bottom order to reflect spatial layout: 1, 2, 3, 4, 5, 6, 7, 14, 11, 12, 13, 8, 9, 10;*
- *We added new figures (Figures 4, 5, and 6) to visualize key metrics;*
- *Figure 4 presents boxplots of omissions per position, using the improved spatial ordering;*
- *We also updated all legends and a supplementary glossary to explain all acronyms clearly (see Supplementary File S1).*

*These revisions ensure the results are easier to interpret and more accessible to readers unfamiliar with our variable naming conventions.*

8. Something I really couldn’t understand is the reason why positions 1,2, and 3 did not result in more omissions than positions 8, 9 and 10. For the maximal lateralization a clear disadvantage for left targets should be present. Overall, for positions 1 to 14 no clear lateralized pattern seems to be visible (a graph would help). This clashes with the conclusions and with the outcome of the clustering. Is this confusion due to the presence of trials with two targets ? From the image it seems as if there were 25 different combinations and I therefore wonder why not using all of them or, in alternative, cluster for left vs right low vs high eccentricity.

**Author Response:** *We appreciate this thoughtful observation and have added a new figure (Figure 4) that visualizes omissions by position and group, which we believe helps to clarify this pattern. As you noted, our data do not show a classic left-right gradient of omission severity. Specifically, positions 1–3 (far left) did not consistently elicit more omissions than positions 8–10 (far right), even in the neglect group.*

*Several explanations may help contextualize this finding:*

- *Neuropsychological evidence (Nikolaenko, 2003 [85]) suggests that right hemisphere lesions—particularly those associated with neglect—disrupt the entire spatial framework used for perception, not just the contralesional field.*
- *When the right hemisphere is impaired, spatial representation is reorganized. Prior studies using irregular raster techniques have shown that individuals begin to rely disproportionately on the central right visual field, rather than the far right or left periphery, as the new attentional anchor. This results in underutilization of both extreme hemifields, which may explain why far-right positions in our study also showed elevated omission rates—even in control patients without neglect.*

*Importantly, our control group consisted of patients with right hemisphere damage but no clinical signs of neglect. Their performance suggests that even in the absence of overt neglect, spatial processing may remain subtly impaired—possibly due to a disruption of global spatial organization. We have revised the Discussion to address this asymmetry more explicitly (lines 701–721) and proposed follow-up studies with neurologically healthy participants and patients with left hemisphere damage to test these hypotheses (lines 854–856).*

9. Clustering: at behavioural it seems unclear which are the variables better predicting neglect. The outcome of the clustering allows to refine the diagnostic threshold to converge more or less closely with the diagnosis of the clinician. Yet I see a circularity issue: if the diagnosis is based on the outcome in paper and pencil tests the outcome which is correctly predicted is exactly what we would like to avoid due to their low sensitivity. As already mentioned, the lack of criteria for describing neglect makes any reference in terms of sensitivity to the paper and pencil tests difficult to be understood.

**Author response:** *Thank you for this insightful comment. We fully agree that the source of diagnostic labels must be independent of the algorithm’s predictors to avoid circular reasoning. To clarify: the diagnosis of visual neglect in our study was not based solely on the Bells Test or Albert’s Test. Rather, it was established by a trained neurologist and neuropsychologist as part of a comprehensive clinical assessment. This included behavioral observation, a qualitative neuropsychological examination following the Lurian approach, functional impairment rating via the Catherine Bergego Scale, and paper-and-pencil tests as supporting instruments. We have now detailed these diagnostic procedures in the Participants section (lines 249–278) to make clear that the clinical diagnosis served as an external criterion, independent from the variables used in the clustering models. The Bells and Albert’s Tests were used post hoc to assess convergent validity, not to label the data for classification. We hope this clarification addresses the concern regarding potential circularity.*

10. The supplementary file contains a wealth of information yet several variables are not explained.

**Author response:** *Thank you for pointing this out. We have now added a new sheet to Supplementary File S1 that provides clear definitions for all variables and acronyms used in the dataset. This should improve clarity and make the supplementary materials fully interpretable for readers.*

11. One paper very similar to the present one is this: <https://www.nature.com/articles/s42003-025-08074-z>

**Author response**: *We appreciate this reference and fully agree on its relevance. We had originally cited the preprint version of this article, but we now reference the final published version throughout the manuscript. It appears as reference [67] in the revised bibliography and is discussed in the Introduction and Discussion in relation to multitasking vulnerability and diagnostic sensitivity in neglect*.

We hope these revisions adequately address your and the reviewers’ concerns. We are grateful for the opportunity to improve the manuscript and believe the updated version presents our findings with greater rigor and transparency.

Thank you once again for your thoughtful consideration.

Sincerely,

Elizaveta V. Vasyura

Lomonosov Moscow State University

vasyuraev@my.msu.ru
