# Supplementary material for: Validation of the Keen Eye computer-based method for diagnosing visual neglect using a dual-task paradigm
Source: PLoS One. 2025 Oct 27;20(10):e0323832. doi: 10.1371/journal.pone.0323832 (PMC12558459; doi:10.1371/journal.pone.0323832)
Supplement: Attachment — Submitted filename: Response_to_Reviewers_auresp_2.docx [file pone.0323832.s006.docx]

**Manuscript PONE-D-25-18591**

**Response to Reviewers**

Dear Dr. Contemori,

We would like to express our heartfelt gratitude to you and the reviewers for your invaluable time and effort in providing such thoughtful and constructive feedback on our manuscript. Your dedication to maintaining the highest scientific standards is deeply appreciated, and the review process has undoubtedly strengthened our work.

In particular, Reviewer #3’s detailed comments regarding the spatial distribution of omissions prompted us to conduct an additional quality-control check. During this process, we discovered an error in the preprocessing of target positions (1 - 14), which affected descriptive statistics, sensitivity/specificity estimates for individual positions, and Figure 4.

Importantly, the core findings of the study - including LSU, RSU, NIL, NIR, LSB, RSB, asymmetry indices, categorical decision analysis, and convergent and criterion validity results - remain fully unchanged. After correcting the preprocessing, the data now display a spatial pattern of omissions that is theoretically expected and consistent with prior literature on visual neglect. This correction makes our conclusions clearer and more robust.

The revision substantially improves consistency with established neuropsychological theories: the previously puzzling ipsilesional omissions (highlighted by Reviewer #3 as atypical) are no longer present, while the expected predominance of contralesional omissions is now clearly observed. In our view, this directly addresses Reviewer #3’s valid concerns and clarifies the interpretation of the Keen Eye method. We value transparency and scientific integrity, and we believe that reporting these corrections is essential to ensure a reliable record. To facilitate verification, we are fully prepared to provide the raw data and preprocessing script upon request.

Additionally, we have made several enhancements to strengthen the manuscript further:

- We have added CBS scores to the S1 supplementary file, enabling direct comparison of psychometric properties (sensitivity and specificity) between the CBS and our proposed Keen Eye method
- We have refined the section heading from "Convergent and criterial validity of the method" to "Psychometric properties of the method" to better reflect the expanded content, including sensitivity statistics comparing our method to traditional paper-and-pencil assessments.

Once again, we extend our sincere appreciation for your continued consideration of our work and for the rigorous peer-review process that has helped us improve this manuscript substantially.

# **Editor’s Comments**

1. If the reviewer comments include a recommendation to cite specific previously published works, please review and evaluate these publications to determine whether they are relevant and should be cited. There is no requirement to cite these works unless the editor has indicated otherwise.

**Authors’ Response:** *We greatly appreciate the reviewers’ suggestions and evaluate each recommendation carefully to ensure it adds genuine value to our work. In this revision, we have included the article recommended by Reviewer #3 in the Discussion section.*

2. Please review your reference list to ensure that it is complete and correct. If you have cited papers that have been retracted, please include the rationale for doing so in the manuscript text, or remove these references and replace them with relevant current references. Any changes to the reference list should be mentioned in the rebuttal letter that accompanies your revised manuscript. If you need to cite a retracted article, indicate the article’s retracted status in the References list and also include a citation and full reference for the retraction notice.

**Authors’ Response:** *In the previous revision, we made the following updates to the reference list:*

- *References 37-41 were added to provide background on the historical development of dual-task paradigms in response to Reviewers.*
- *Reference 59 was corrected due to an earlier citation error: Zhou L, Zhen Z, Liu J, Zhou K. Neural mechanisms underlying individual differences in attentional blink. J Vis. 2019;19(10):108. doi:10.1167/19.10.108.*
- *Reference 67 was updated to replace a preprint with the final published article: Blini E, D'Imperio D, Romeo Z, De Filippo De Grazia M, Passarini L, Pilosio C, Meneghello F, Bonato M, Zorzi M. Susceptibility to multitasking in stroke is associated to multiple-demand system damage and leads to lateralized visuospatial deficits. Commun Biol. 2025 May 12;8(1):734. doi: 10.1038/s42003-025-08074-z.*
- *References 68-70, 72 were not deleted but were relocated to another section, leading to a renumbering.*
- *Reference 80 (Blini et al., 2016) was removed because the current study does not address right-sided spatial neglect, and this citation was no longer relevant to our sample of exclusively right-hemisphere patients.*

*In this revision, the only addition to the reference list is Reference 89 (Bonato et al., 2019), included following Reviewer #3’s recommendation. No other changes were made.*

## **Reviewers' Comments to the Authors:**

**Reviewer #1**

As far as my points are concerned, I think that the authors responded very well. I would endorse this paper for publication.

Only one minor note for the authors: Please consider revising the title again: I don’t think it is necessary to specify “in dual-task paradigm” since this is what the Keen eye method is, but if you really want to keep it consider adding an article as in “in A dual-task paradigm” or perhaps “using a dual-task paradigm”.

Best wishes

**Authors’ Response:** *Thank you very much for your positive evaluation and for this helpful stylistic recommendation. We have revised the title and now use “using a dual-task paradigm” as suggested. We appreciate your feedback, which helped further improve the clarity of the manuscript.*

**Reviewer #2**

Reviewer #2: I thank the authors for their thorough and thoughtful revisions. All my previous comments have been addressed in a satisfactory manner, and the manuscript has been significantly improved.

One additional recommendation concerns the use of the term "diagnostic". While the Keen Eye method shows promising classification accuracy based on data-driven thresholds, I would advise caution in referring to it as a "diagnostic tool" in a strict clinical sense, at least until standardization procedures (e.g., normative scoring based on healthy or diverse clinical populations) are completed. A brief clarification of this point in the Discussion would be appropriate and would strengthen the positioning of the method as a potentially diagnostic instrument, pending further validation.

In summary, I have no further mandatory requests. I support the publication of this manuscript, with the optional recommendation above.

***Authors’ Response:*** *Thank you for your supportive evaluation. We fully agree with your comment regarding the cautious use of the term “diagnostic tool.” In the revised manuscript, we added a clarification in the Limitations section (lines 925-930). Although the Keen Eye method demonstrates high classification accuracy based on data-driven thresholds, these criteria require further validation through standardization procedures, including the collection of normative data from healthy participants. This step is essential to ensure that predictive models do not rely solely on data from clinical samples.*

*We appreciate your insightful recommendation, which has strengthened the methodological positioning of our study.*

**Reviewer #3**

Reviewer #3:

1. I thank the authors for having revised the manuscript according to my comments. While I think the strength of this interesting study is significantly improved, I also noticed the interpretation of the findings seems to be still ambiguous. I better detail below this only major point remaining (point 4. of previous review) which in my view needs to be addressed by both the framing of the study as well as by describing and interpreting the data better.

**Authors’ Response:** *Thank you very much for this observation. We found the spatial omission pattern shown in the descriptive statistics and Figure 4 difficult and unexpected. Your renewed attention to the unsatisfactory nature of our previous explanation prompted us to perform an additional quality-control check, during which we discovered an error in preprocessing the individual target positions (1-14). Correcting this error changed the results related to individual target positions but did not affect the other indices of the method or the categorical decision analysis. We are grateful that you drew attention to this issue, which allowed us to substantially improve the accuracy and clarity of our data interpretation.*

2. Framing: Authors explicitly mention the clinical use of the task and should be commended in case their choice is to provide it openly to the neuropsychological community. Yet, expanding on point 4. of the first revision it still seems rather unclear whether the final aim is to achieve the same sensitivity of standard tests (paper and pencil + CBS) or whether this computer-based approach can result in different/better sensitivity. This is crucial as someone might claim that trying to replicate the (low) diagnostic sensitivity characterizing paper and pencil tests is not an optimal objective. I suggest that the reference to this aspect should be more explicit and I also suggest explaining more in depth what the task is actually measuring.

**Authors’ Response:** *Thank you for this valuable comment. We explicitly clarified in the Introduction that our aim is to exceed the sensitivity of classical paper-and-pencil and CBS tests rather than simply replicate it (lines 223-227). We also provided a brief description of what the Keen Eye method is designed to measure. To substantiate this aim, we calculated the sensitivity of standard paper-and-pencil tests and CBS and directly compared them with Keen Eye performance (lines 671-677).*

3. This seems mandatory also when considering the very peculiar spatial distribution of omissions (Fig. 4), which seem to be more ipsilesional than contralesional.

**Authors’ Response:** *This issue has been corrected following the preprocessing revision. The updated results now clearly show that patients with visual neglect predominantly omit contralesional stimuli and detect ipsilesional stimuli more reliably, consistent with classical descriptions of neglect.*

4. Data description and interpretation: From the above mentioned point of view It would be clearer if the % of ipsi vs contra lesional omissions for graphs 5 would be spelled out.

**Authors’ Response:** *We agree. To more accurately represent spatial bias, we reconstructed Figures 4 and 5 using the percentage of omissions rather than raw counts. This adjustment accounts for the fact that unilateral and bilateral presentations are not equally frequent and that certain positions (2, 5, 7, 14, 12, 9) occur six times whereas others occur nine times. The new figures now display the percentage of missed targets relative to the maximum possible omissions for each variable, improving clarity and comparability.*

5. I would rather tend to think that the peculiar asymmetry described which favour the contralesional hemispace is due to compensation. I might be wrong but it seems prominent in patients classified as being without VN. For reference about the spatial distribution of ipsilesional omissions at different ipsilesional eccentricities authors might want to consult the study with ipsilesional targets presented with low and high eccentricity by Bonato, Romeo et al., 2019 (Frontiers). Also the asymmetries represented in figure 6 should be in my view described as indexing a bias towards ipsi or contralesional space (now this crucial aspect is not clear, in particular when considering figure 4).

**Authors’ Response:** *Thank you for pointing this out. To quantify group differences, we compared asymmetry coefficients between patients with neglect and those without, and we additionally performed paired Wilcoxon tests comparing right- versus left-sided omissions within each group (lines 490-502). The results show a clear contralesional bias in the neglect group and only a mild bias in the control group.*

*We also consulted the study by Bonato et al. (2019), which confirmed that our corrected results align with previously reported spatial attention patterns.*

*Finally, we revised the description of Figure 6 to explicitly state that these indices reflect spatial bias toward either ipsilesional or contralesional hemispace, improving interpretability.*

MINOR

6. The description of the analysis is sometimes a bit more difficult than the standard. I suggest explaining the rationale/meaning more explicitly. The comprehension seems sometimes difficult also because the amount of acronyms is still overwhelming.

**Authors’ Response:** *We fully understand your concern regarding the large number of variables and acronyms. This comprehensive set of measures was intentionally designed to enable the most detailed analysis of the methodology’s performance. In the future, as part of standardization, it may be appropriate to limit the number of variables to those with the highest prognostic value. To improve clarity in the current manuscript, we added explicit explanations for the rationale and interpretation of key variables (lines 400-408, 420-422, 427-428, 435-436, 440-443)*

7. I suggest avoiding colloquial terms like “skeleton” for describing the spatial distribution of attention.

**Authors’ Response:** *Thank you for this note. We replaced the term “skeleton” with the more precise phrase “attentional structure” (line 768).*

8. For neuropsychological standards the sample is rather wide, I would emphasize this strength better.

**Authors’ Response:** *We agree. This important strength of our study is now explicitly highlighted in the Discussion (lines 736-740).*

9. In the abstract as well as in the text target positions are referred to as “fixed”. I suggest avoiding this term as it can be easily misunderstood. Rather, I would better emphasize that the position of appearance was variable… and eventually (but in the methods only) that the targets appeared in a fixed order. The random (fixed) position of appearance is a major strength of this study along with the vertical measure. I would highlight it better as spatial uncertainty might trigger interesting mechanisms including the one I see here resulting in better contralesional performance (was this statistically tested?) which I interpret as compensatory.

**Authors’ Response:** *Thank you for pointing this out. We revised the abstract and main text to clarify that target positions varied across trials, explicitly noting in the Methods section that they were presented in a predetermined sequence (lines 692-694, Figure 2 caption). Regarding whether better contralesional performance was statistically tested: after correcting the preprocessing of target positions, the data now demonstrate the expected predominance of contralesional omissions, which we confirmed using a Wilcoxon test.*

10. I suggest checking for typos and grammatical errors.

**Authors’ Response:** *Thank you for this reminder. We corrected all detected typos, including those in Table 3.*

Thank you once again for your thoughtful consideration. Your comments substantially improved both the clarity and theoretical consistency of our manuscript.

Sincerely,

Elizaveta V. Vasyura

Lomonosov Moscow State University

vasyuraev@my.msu.ru
