# Supplementary material for: Validation of the Keen Eye computer-based method for diagnosing visual neglect using a dual-task paradigm
Source: PLoS One. 2025 Oct 27;20(10):e0323832. doi: 10.1371/journal.pone.0323832 (PMC12558459; doi:10.1371/journal.pone.0323832)
Supplement: Attachment — Submitted filename: Response_to_Reviewers_auresp_3.docx [file pone.0323832.s007.docx]

**Manuscript PONE-D-25-18591**

**Response to Reviewers**

Dear Dr. Contemori,

We would like to sincerely thank you and the reviewers for the careful evaluation of our manuscript and for the constructive feedback provided at each stage of the review process. We greatly appreciate the opportunity to further revise our work. Below we provide a detailed response to all points raised. All changes in the manuscript are highlighted in the revised version with track changes.

## **Editor’s Comments**

If the reviewer comments include a recommendation to cite specific previously published works, please review and evaluate these publications to determine whether they are relevant and should be cited. There is no requirement to cite these works unless the editor has indicated otherwise.

Please review your reference list to ensure that it is complete and correct. If you have cited papers that have been retracted, please include the rationale for doing so in the manuscript text, or remove these references and replace them with relevant current references. Any changes to the reference list should be mentioned in the rebuttal letter that accompanies your revised manuscript. If you need to cite a retracted article, indicate the article’s retracted status in the References list and also include a citation and full reference for the retraction notice.

***Author response:*** *We carefully reviewed our reference list and confirmed that it contains no retracted papers. As requested, we also checked all references for accuracy and updated formatting where needed. One additional citation had already been included in the previous round per reviewer recommendation. During this comprehensive review, we identified and corrected several formatting inconsistencies to ensure compliance with PLOS Vancouver style requirements:*

- *Reference 14: Corrected the formatting of the book chapter citation to properly include "In:" before the editor name, appropriate punctuation, and "pp." for page ranges (Harvey M. Perspectives on visuospatial neglect).*
- *Reference 22: Updated the encyclopedia chapter formatting to use consistent page numbering format "pp. 3582-3583" instead of abbreviated form (Heilman KM, Lamb D, Williamson JB. Vertical Neglect).*
- *Reference 44: Corrected a typographical error in the publication year from "2917" to "2017" (Lebed AA, Korovkin SY. The unconscious nature of insight).*
- *Reference 47: Improved the preprint citation formatting by standardizing the month format to "March" and ensuring proper spacing around the DOI (Halder S, Raya DV, Sridharan D. Distinct neural bases of subcomponents of the attentional blink).*
- *Reference 69: Added the missing period at the end of the citation (Bonato M, Priftis K, Marenzi R, Umiltà C, Zorzi M. Increased attentional demands impair contralesional space awareness following stroke).*
- *Reference 83: Added the appropriate DOI for completeness (Azouvi P, Samuel C, Louis-Dreyfus A, et al. Sensitivity of clinical and behavioral tests of spatial neglect after right hemisphere stroke).*
- *Reference 99: Standardized the preprint citation formatting by spelling out "September" fully and correcting the citation date format (Dietz MJ, Nielsen JF, Roepstorff A, Garrido MI. Dysconnection of right parietal and frontal cortex in neglect syndrome).*

*All corrections ensure consistency with PLOS formatting requirements and improve the overall quality and accuracy of our reference list.*

## **Reviewers' Comments to the Authors:**

**Reviewer #1**

I have assessed the differences introduced in the last review round to the best of my possibilities and think that the results are sound. I commend the authors for self-correcting their work.

***Author response:*** *We sincerely thank you for your supportive evaluation and kind words.*

**Reviewer #2**

All my previous comments have been addressed in a satisfactory manner and I would endorse this paper for publication.

***Author Response:*** *Thank you very much for your endorsement and encouraging feedback.*

**Reviewer #3**

1. I am happy to read that my comments on the atypical spatial distribution of omissions eventually led to discover and correct an error in the data coding. Now results in general and more specifically graph 4 makes much more sense and also the rationale seems more convincing.

***Author response:*** *We are especially grateful for your thorough feedback, which has significantly improved the clarity and rigor of our work. Below we address your remaining minor points in detail.*

Some minor and final comments starting from graphs:

2. I could not find figure captions and therefore the values represented by the histogram seem a bit obscure. (Figure 4) Is the black line a median/average value ? Is so, does the histogram represent (Yaxis) “percentage of maximum omissions” i.e. the worst individual performance for that location ? If so please be explicit as it seems a non-standard statistic to report.

**Author response:** *Thank you for pointing this out. We have revised the captions for Figures 4–6 (lines 485-497) to explicitly clarify:*

- *the black horizontal line inside each box indicates the median,*
- *the Y-axis represents Percentage of Maximum Possible Omissions (%), calculated as the number of missed targets relative to the maximum number of presentations for each stimulus position,*
- *the normalization was necessary because not all positions were presented equally often (e.g., positions 2, 5, 7, 9, 12, and 14 appeared six times; all others appeared nine times),*
- *we also added outliers and explicitly noted that outliers are represented by dots.*

3. Ipsilesional omissions: I no longer see the need to discuss in depth this aspect unless there is a significant difference (maybe I missed it). My request to tackle this issue was related to the previous pattern. Now that data have been recoded and ipsilesional omissions are not particularly evident I would avoid insisting on this topic. More specifically, the lack of a control group of healthy participants does not allow to conclude much. If anything, I would instead discuss more in depth the contralesional omissions found in the N- group, as that is a result of great clinical importance.

***Author response:*** *We agree. We have streamlined the discussion by removing extended speculation on ipsilesional omissions and shifted the focus toward the clinical relevance of contralesional omissions in patients without neglect (lines 759-778). We emphasize that even in this group, subtle left-sided deficits were detectable.*

4. I would also consider calling the current “control group” something more informative like “patients without neglect diagnosis”. Regardless on the specific collective name I would avoid referring to them as “control participants” as the readers might think they were healthy controls.

***Author response:*** *Thank you for this helpful suggestion. We have replaced the term control group throughout the manuscript with patients without neglect diagnosis. We believe this improves clarity for the reader.*

We are deeply grateful for the constructive guidance from the reviewers and editorial team. We believe these final adjustments have further strengthened the clarity and clinical significance of our work.

Sincerely,

Elizaveta V. Vasyura

Lomonosov Moscow State University

vasyuraev@my.msu.ru
